# Plasma leptin level is associated with cardiac autonomic dysfunction in patients with type 2 diabetes: HSCAA study

**DOI:** 10.1186/s12933-015-0280-6

**Published:** 2015-09-04

**Authors:** Masafumi Kurajoh, Hidenori Koyama, Manabu Kadoya, Mariko Naka, Akio Miyoshi, Akinori Kanzaki, Miki Kakutani-Hatayama, Hirokazu Okazaki, Takuhito Shoji, Yuji Moriwaki, Tetsuya Yamamoto, Masanori Emoto, Masaaki Inaba, Mitsuyoshi Namba

**Affiliations:** Division of Diabetes, Endocrinology and Metabolism, Department of Internal Medicine, Hyogo College of Medicine, 1-1 Mukogawa-cho, Nishinomiya, Hyogo 663-8501 Japan; Department of Endocrinology, Metabolism and Molecular Medicine, Osaka City University Graduate School of Medicine, Osaka, Japan

**Keywords:** Leptin, Heart rate variability, Cardiac autonomic dysfunction, Type 2 diabetes, Visceral fat accumulation

## Abstract

**Background:**

It has been shown that visceral fat accumulation is associated with autonomic dysfunction, though the precise mechanism remains unclear. A recent basic study found that leptin can directly modulate autonomic function through the dorsomedial hypothalamus in relation to obesity. Here, we investigated the mutual relationships among plasma leptin, visceral fat accumulation, and cardiac autonomic dysfunction in patients with type 2 diabetes.

**Methods:**

This cross-sectional study included 100 diabetic patients, and 100 age- and gender-matched non-diabetic patients with cardiovascular risk factors. Plasma leptin and soluble leptin receptor levels, visceral fat area (VFA), and heart rate variability (HRV) were determined in addition to classical cardiovascular risk factors.

**Results:**

In the type 2 diabetic patients, VFA was significantly (p < 0.05) and inversely associated with HRV parameters (SDNN: r = −0.243; SDANN5: r = −0.238), while the plasma level of leptin, but not soluble leptin receptor, was also significantly (p < 0.05) and inversely associated with HRV parameters (SDNN: r = −0.243; SDANN5: r = −0.231). Multiple regression analysis showed that plasma leptin was significantly associated with SDNN and SDANN5 independent of other factors, including age, gender, presence of hypertension and dyslipidemia, duration of diabetes, HbA1c, and eGFR. Furthermore, the relationship of leptin with SDNN and SDANN5 (β = −0.279 and −0.254, respectively) remained significant (p < 0.05) after adjustment for VFA. In patients without diabetes, no significant associations were observed between leptin and any of the HRV parameters.

**Conclusions:**

Hyperleptinemia may be involved in cardiac autonomic dysfunction in patients with type 2 diabetes and visceral obesity.

## Background

The prevalence of obesity has been increasing throughout the world over the past several decades to become a global health problem [1]. Excess visceral fat accumulation accompanied by obesity contributes to development of hypertension, diabetes, dyslipidemia, and cardiovascular events [2, 3]. Adiposity and cardiac autonomic dysfunction are closely associated [4], which may be involved in a higher incidence of cardiovascular events in obese individuals [5, 6]. However, the underlying mechanisms for cardiac autonomic dysfunction related to obesity remain unclear.

Leptin, a 16-kDa peptide hormone mainly derived from adipose tissue, suppresses appetite and increases energy expenditure via hypothalamic neurons expressing the receptor for leptin (Ob-R) [7, 8]. In clinical settings, plasma leptin concentrations have found to be strongly and positively correlated to adiposity [9–11], suggesting that high leptin levels lose their ability to restrain feeding and fat accumulation in obesity. Of importance, a recent study clearly showed that hyperleptinemia, a result of obesity, directly modulated blood pressure and heart rate by acting on the dorsomedial hypothalamus expressing Ob-R in obese mice, probably via the sympathetic nervous system [12].

These basic findings led us to examine whether plasma leptin contributes to the pathophysiology of autonomic dysfunction in obesity in humans. Measuring heart rate variability (HRV) using standard electrocardiographic monitoring is a practical way to assess impaired autonomic nervous system in clinical settings [13]. Reduced HRV predicts all-cause mortality and cardiovascular events not only in community-based population [5, 6], patients with myocardial infarction [14], and in diabetic patients [15–17]. Potential mechanisms for these associations include higher corrected QT prolongation, low exercise capacity, higher levels of inflammatory biomarker, and deregulation of progenitor cells [18–20]. Thus far, no reports examining the associations among adiposity, plasma leptin, and HRV in patients with type 2 diabetes have been presented. In the present study, we examined the mutual relationships among plasma leptin, HRV, and visceral fat volume in 100 patients with diabetes, as well as 100 age- and gender-matched non-diabetic patients with cardiovascular risk factors.

## Methods

### Study design and participants

This cross-sectional study included 100 patients with type 2 diabetes, and 100 age- and gender-matched patients without diabetes who participated in the Hyogo Sleep Cardio-Autonomic Atherosclerosis (HSCAA) Study, which was designed to examine the impacts of sleep, cardiac autonomic dysfunction, and subclinical atherosclerosis on cardiovascular events. All patients agreed to participate in the study by providing written informed consent and the study was approved by the Ethics Committee of Hyogo College of Medicine (approval No. 948). From the cohort, initial 100 sequential diabetic patients were included, and corresponding age- and gender-matched non-diabetic patients were sequentially selected. Details of the cohort appear elsewhere [21], and classical cardiovascular risk factors and diabetes were evaluated as described in that publication. In diabetic groups, use of anti-diabetic agents included sulfonylureas (n = 30), metformin (n = 35), pioglitazone (n = 11), dipeptidyl peptidase 4 (DPP4) inhibitors (n = 33), and α-glucosidase inhibitors (n = 9), glucagon like peptide 1 (GLP1) analogues (n = 2), and insulins (n = 19). Past history of cardiovascular disease (CVD) was defined as the presence of coronary artery disease (medical history of myocardial infarction or revascularization), cerebrovascular disease, or peripheral arterial disease (PAD). Diabetic neuropathy was defined as the presence of two or more of the following: neuropathic symptoms, decreased distal sensation, and unequivocally decreased or absent ankle reflexes [22]. Serum creatinine was measured using the enzymatic method. Estimated glomerular filtration rate (eGFR) in each patient was calculated using an equation for Japanese subjects, as previously described [23]. This cross-sectional study excluded participants with chronic kidney disease (CKD), defined as eGFR less than 30 ml/min/1.73 m^2^. The level of urinary albumin excretion (UAE) was measured in 24-h urine specimens by immunoturbidimetry. UAE <30, 30–300, and >300 mg/day was defined as normo-, micro-, and macro-albuminuria, respectively.

### Cardiac autonomic nervous function

To evaluate cardiac autonomic function, HRV was determined using an Active Tracer (AC-301A, Arm Electronics, Tokyo, Japan), as previously described [24]. According to the recommendations for clinical use of HRV [13], the standard deviation of the NN(RR) interval (SDNN) and standard deviation of the average NN(RR) intervals for each 5-min (SDANN5) period were calculated. SDNN was considered to reflect all cyclic components responsible for variability in heart rate, while SDANN5 was used to represent change in heart rate due to cycles longer than 5 min [13].

### Plasma leptin and soluble leptin receptor

Blood samples were obtained essentially in the morning after an overnight fast during measurement of HRV and then quickly centrifuged to obtain plasma. Plasma leptin and soluble Ob-R (sOb-R) levels were measured using an enzyme-linked immunosorbent assay kit (R&D Systems, Inc., Minneapolis, MN, USA), as previously described [25]. The intra- and inter-assay coefficients of variation for leptin were 3.2 and 4.9 %, respectively, and those for sOb-R were 3.5 and 6.7 %, respectively.

### Measurement of visceral and subcutaneous fat area

Using abdominal computed tomography (SOMATOM Definition, Siemens Medical Solutions, Forchheim, Germany), we acquired a single 10-mm slice at the level of the umbilicus. Visceral fat area (VFA) and subcutaneous fat area (SFA) values were calculated with a Ziostation workstation (Ziosoft Inc., Tokyo, Japan), as previously described [26].

### Statistical analysis

For analysis, HRV parameters (SDNN, SDANN5), and plasma leptin and sOb-R levels were logarithm-transformed (log) to achieve normal distribution. To compare variables between patients with and without diabetes, a non-repeated *t* test (continuous variables with normal distribution) and Chi square test (for categorical variables) were utilized. We performed simple and multiple regression analyses to evaluate the relationships between HRV parameters and various clinical parameters, including plasma leptin or sOb-R, and VFA. In multiple regression analysis, HRV parameters were set as a dependent variables, and VFA and plasma leptin levels were set as independent variables after adjusting for age, gender, eGFR, HbA1c levels, duration of diabetes (in affected patients), and presence of hypertension and dyslipidemia. All statistical analyses were performed by using the Statistical Package for the Social Sciences software (PASW Statistics version 18.0). All reported p values are 2-tailed and were considered statistically significant at a level <0.05.

## Results

### Clinical characteristics of subjects with and without diabetes

The subject characteristics are shown in Table 1. Those with diabetes exhibited significantly higher body mass index (BMI), eGFR, fasting plasma glucose (FPG), HbA1c, VFA, and SFA values. The prevalence of hypertension and dyslipidemia tended to be higher in diabetic than non-diabetic patients, while plasma leptin and sOb-R levels, and HRV parameters were not significantly different between the groups.

**Table 1:** Clinical characteristics of patients with and without diabetes

Variables	DM (n = 100)	NDM (n = 100)	p value
Age (years)	62.9 ± 9.3	62.9 ± 9.5	0.988
Gender (male/female)	62/38	62/38	
BMI (kg/m^2^)	26.1 ± 4.7	23.3 ± 3.3	<0.001
Hypertension (yes/no)	71/29	59/41	0.075
Dyslipidemia (yes/no)	72/28	59/41	0.053
Past CVD events (yes/no)	21/79	17/83	0.762
Smoker (yes/no)	32/68	20/80	0.053
eGFR (ml/min/1.73 m^2^)	85.0 ± 24.9	76.4 ± 16.0	0.004
FPG (mg/dl)	132.9 ± 36.7	94.5 ± 10.4	<0.001
HbA1c (%)	7.2 ± 1.3	5.3 ± 0.4	<0.001
Duration of DM (years)	10.4 ± 9.0		
Diabetic neuropathy (yes/no)	48/52		
Diabetic nephropathy (normo-/micro-/macro-)	78/19/3		
Anti-diabetes agents			
Sulfonylurea (%)	30		
Metformin (%)	35		
Pioglitazone (%)	11		
DPP4 inhibitor (%)	33		
α-glucosidase inhibitor (%)	9		
GLP1 analogue (%)	2		
Insulin (%)	19		
VFA (cm^2^)	105.2 ± 47.9	78.3 ± 44.6	<0.001
SFA (cm^2^)	165.3 ± 93.1	129.8 ± 74.1	0.003
log (leptin) (ng/mL)	0.81 ± 0.41	0.73 ± 0.41	0.166
log (sOb-R) (ng/mL)	1.35 ± 0.13	1.33 ± 0.14	0.214
log (SDNN) (ms)	2.02 ± 0.14	2.05 ± 0.12	0.184
log (SDANN5) (ms)	1.97 ± 0.14	1.99 ± 0.13	0.309

Data are expressed as the mean ± standard deviation and number for dichotomous variables

Plasma leptin and sOb-R levels, and all HRV parameters (CVRR, SDNN, SDANN5, HRV triangular index) were natural logarithm-transformed (log) to achieve a normal distribution. P values shown are for comparisons of the means of 2 groups (unrepeated t-test) or percentages (Chi square test)

*BMI* body mass index, *CVD* cardiovascular disease, *eGFR* estimated glomerular filtration rate, *FPG* fasting plasma glucose, *DM* diabetes mellitus, *DPP4* dipeptidyl peptidase 4, *GLP1* glucagon-like peptide 1, *VFA* visceral fat area, *SFA* subcutaneous fat area, *normo-* normal albuminuria, *micro-* microalbuminuria, *macro-* macro-albuminuria, *sOb-R* soluble leptin receptor, *SDNN* standard deviation of NN(RR) interval, *SDANN5* standard deviation of average NN(RR) intervals for each 5-min period

### Associations of VFA, SFA, plasma leptin, and sOb-R levels with HRV parameters

**Fig. 1:**
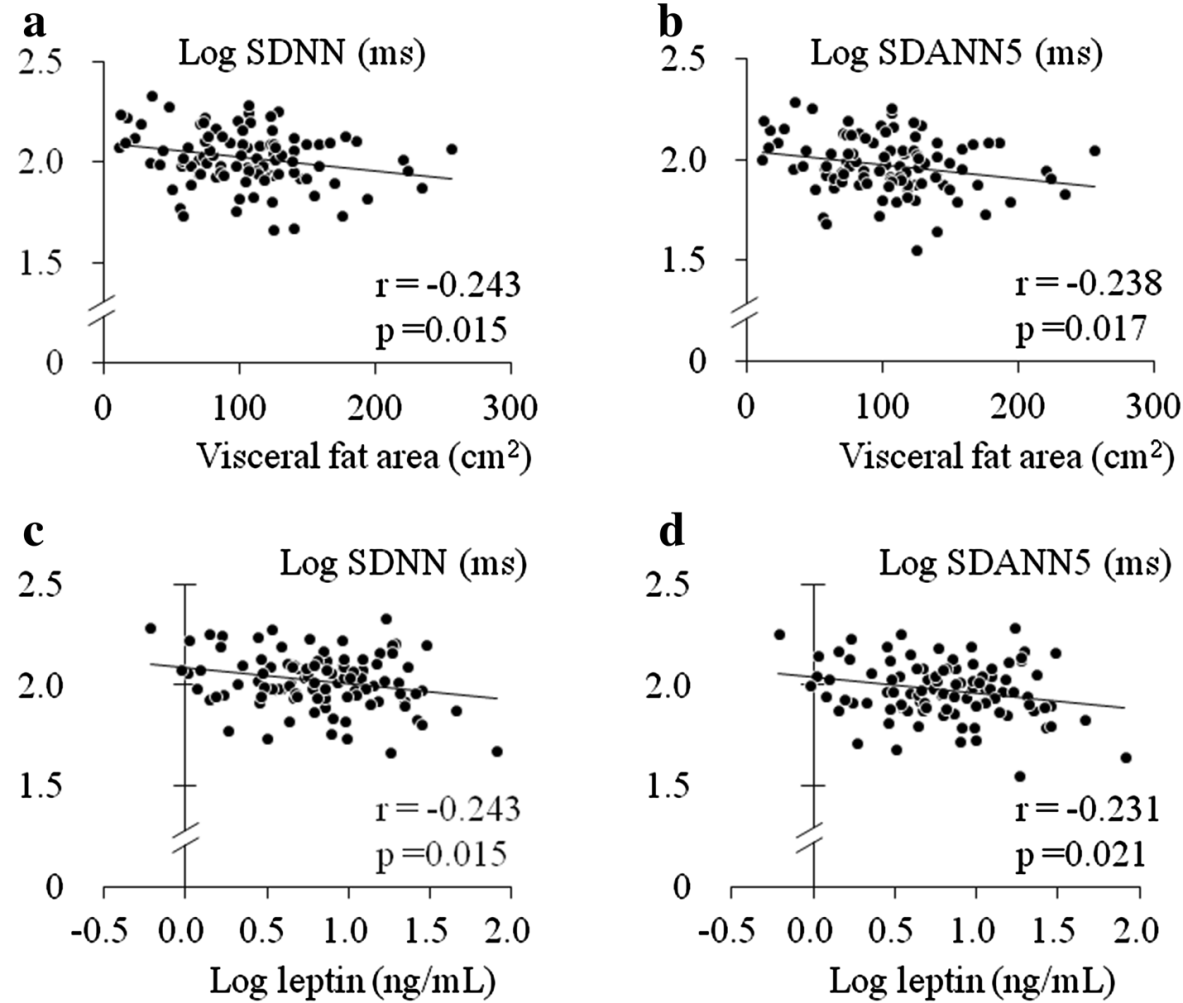
Associations of HRV parameters (**a**, **c** SDNN, **b**, **d** SDANN5) with visceral fat area (**a**, **b**) and plasma leptin levels (**c**, **d**) in patients with type 2 diabetes

Table 2 shows the correlation coefficients between VFA, SFA, plasma leptin and sOb-R levels, and HRV parameters. VFA, but not SFA, was significantly and inversely associated with HRV parameters in patients with diabetes (Fig. 1a, b; Table 2). In contrast, such relationships were not observed in the non-diabetic patients (Table 2). Plasma leptin level was significantly and inversely associated with SDNN and SDANN5 in diabetic patients (Fig. 1c, d; Table 2), but not in the non-diabetic group (Table 2). Plasma sOb-R level was not significantly associated with HRV parameters in either group. On the other hand, plasma leptin levels were significantly and positively associated with VFA (r = 0.417, p < 0.001, r = 0.262; p = 0.008) and SFA (r = 0.732, p < 0.001, r = 0.693; p < 0.001) in both the diabetic and non-diabetic subjects. Also, plasma sOb-R level showed a tendency to be associated with VFA (r = −0.181, p = 0.072, r = −0.190; p = 0.058), but not with SFA, in both groups.

**Table 2:** Associations of VFA, SFA, plasma leptin, and sOb-R levels with HRV parameters

	DM (n = 100)				NDM (n = 100)			
	log (SDNN)		log (SDANN5)		log (SDNN)		log (SDANN5)	
	r	p	r	p	r	p	r	p
VFA	−0.243	0.015	−0.238	0.017	−0.039	0.699	−0.001	0.993
SFA	−0.116	0.252	−0.113	0.261	0.053	0.599	0.078	0.438
log (leptin)	−0.243	0.015	−0.231	0.021	−0.020	0.842	0.005	0.959
log (sOb-R)	−0.061	0.545	−0.040	0.694	0.106	0.296	0.103	0.307

HRV parameters (SDNN, SDANN5), and plasma leptin and sOb-R levels were natural logarithm-transformed (log) to achieve a normal distribution

*HRV* heart rate variability, *SDNN* standard deviation of NN(RR) interval, *SDANN5* standard deviation of average NN(RR) intervals for each 5-min period, *VFA* visceral fat area, *SFA* subcutaneous fat area, *sOb-R* soluble leptin receptor

### Associations of plasma leptin levels with HRV parameters in patients with or without diabetic neuropathy

Plasma leptin (0.83  ±  0.41 vs. 0.79  ±  0.42, p = 0.610), SDNN (2.00  ±  0.13 vs. 2.04  ±  0.14, p = 0.165), and SDANN5 (1.96  ±  0.13 vs. 1.99  ±  0.14, p = 0.183) were not significantly different when compared between diabetic patients with (n = 48) and without (n = 52) neuropathy. In patients with diabetic neuropathy, leptin was significantly associated with SDNN (r = −0.323, p = 0.02) or SDANN5 (r = −0.338, p = 0.014). On the other hand, in patients without diabetic neuropathy, leptin was not significantly associated with SDNN (r = −0.137, p = 0.355) or SDANN5 (r = −0.092, p = 0.534).

### Plasma leptin level is associated with HRV parameters independent of clinical factors and adiposity in diabetic patients

To further examine whether the relationship of plasma leptin with cardiac autonomic dysfunction in diabetic patients was independent of the other confounding factors, multiple regression analyses were performed (Table 3). In basic model 1, which included age, male gender, eGFR, HbA1c, presence of hypertension and dyslipidemia, and duration of diabetes as covariates, duration of diabetes was the sole significant factor associated with SDNN and SDANN5. When VFA was included as an additional covariate (model 2), both duration of diabetes and VFA were significantly associated with HRV parameters. In model 3 (addition of plasma leptin to model 1 covariates), plasma leptin remained significantly associated with SDNN and SDANN5. When both VFA and plasma leptin were added to the model 1 covariates (model 4), plasma leptin, but not VFA, exhibited significant and independent associations with SDNN and SDANN5. When BMI was included as an additional covariate to model 4, plasma leptin, but neither VFA nor BMI, remained significantly associated with SDNN and SDANN5 (data no shown). When use of any of an anti-diabetic agent (sulfonylurea, metformin, pioglitazone, DPP4 inhibitor, α-glucosidase inhibitor, GLP1 analogue, or insulin) was included as an additional covariate to model 4, plasma leptin remained significantly associated with SDNN (data no shown). Among the agents, only use of insulin was independently associated with SDNN (β = −0.224, p = 0.034) together with plasma leptin (β = −0.256, p = 0.042) and duration of diabetes (β = −0.223, p = 0.041). Finally, when presence of previous CVD events was included as an additional covariate to model 4, plasma leptin remained significantly associated with SDNN (β = −0.275, p = 0.033) and tended to associated with SDANN5 (β = −0.243, p = 0.061). When multiple regression analyses were re-performed in 79 non-CVD patients (model 4), both plasma leptin and duration of diabetes showed tendency to associate with SDNN (β = −0.280, p = 0.062; β = −0.233, p = 0.051, respectively) and SDANN5 (β = −0.281, p = 0.066; β = −0.207, p = 0.087, respectively). In contrast, neither VFA nor plasma leptin was independently associated with HRV parameters in the non-diabetic patients (data not shown).

**Table 3:** Multiple regression analysis of factors associated with HRV parameters in 100 patients with diabetes

Variables	log (SDNN)				log (SDANN5)			
	Model 1	Model 2	Model 3	Model 4	Model 1	Model 2	Model 3	Model 4
Age	−0.187	−0.170	−0.215	−0.204	−0.166	−0.149	−0.193	−0.180
Gender (female = 0, male = 1)	−0.040	0.005	−0.190	−0.146	−0.038	0.006	−0.177	−0.132
Hypertension (absence = 0, presence = 1)	−0.103	−0.040	−0.023	−0.010	−0.119	−0.059	−0.044	−0.031
Dyslipidemia (absence = 0, presence = 1)	−0.136	−0.127	−0.110	−0.111	−0.141	−0.133	−0.117	−0.118
eGFR	−0.140	−0.118	−0.152	−0.141	−0.106	−0.085	−0.118	−0.106
HbA1c	−0.117	−0.071	−0.096	−0.080	−0.096	−0.051	−0.076	−0.059
Duration of DM	−0.250^†^	−0.286^†^	−0.264^†^	−0.277^†^	−0.244^†^	−0.278^†^	−0.257^†^	−0.270^†^
VFA		−0.233^†^		−0.096		−0.227^†^		−0.101
log (leptin)			−0.333^†^	−0.279^†^			−0.312^†^	−0.254^†^
Adjusted r^2^	0.100	0.140	0.179	0.176	0.089	0.126	0.156	0.153
p value	0.018	0.005	0.001	0.001	0.028	0.008	0.002	0.004

*eGFR* estimated glomerular filtration rate, *DM* diabetes mellitus, *VFA* visceral fat area, *SDNN* standard deviation of NN(RR) interval, *SDANN5* standard deviation of average NN(RR) intervals for each 5-min period, *HRV* heart rate variability

^†^Denotes p value <0.05

## Discussion

### Plasma leptin in obesity and diabetes

Plasma leptin levels are closely associated with adiposity [9–11], and its level correlated with increased metabolic syndrome components [27]. Plasma leptin level in diabetes is rather controversial; one recent study does not observe any significant difference between diabetic and non-diabetic subjects [28], others reported significant lower level in type 1 diabetic subjects [29] and type 2 diabetics with similar adiposity [30]. In our current study, plasma leptin level was comparable between diabetic and non-diabetic patients even though BMI, VFA and SFA are significant higher in diabetes. These controversial findings may be due to the status of insulin resistance and deficiency since plasma leptin level may be modified by insulin or C-peptide [29, 31].

### Leptin and autonomic function

Recent study had shown that leptin may directly modulate autonomic function in obese mouse [12]. However, limited studies have shown an association of plasma leptin level with HRV in humans. In healthy subjects or police officers, short term frequency-domains of HRV are associated with plasma leptin [32, 33]. The relationships of time-domains of HRV, markers of overall autonomic function, with plasma leptin are only shown in male school teachers [34] and males with acute myocardial infarction [35]. Only a limited study showed significant association of HRV parameters with adiponectin/leptin ratio in diabetic subjects [36]. The present study is the first to show its relationship in type 2 diabetic patients, with the relationship fully adjusted for clinical parameters including quantitatively determined visceral adiposity. Its association was specific to diabetic patients since no significant associations were observed between leptin and any of the HRV parameters in non-diabetic patients.

### Potential mechanisms why association between plasma leptin and HRV observed in diabetic patients only

Although it is not clear at present why the independent relationship between plasma leptin and HRV was observed in diabetic patients only, the presence of high glucose level might be a potential reason. Leptin activates proopiomelanocortin (POMC) neuron through the stimulation of the janus kinase 2-phosphoinositide 3-kinase pathway in POMC neuron and the inhibition of gamma-aminobutyric acid (GABA) release to POMC neuron in neuropeptide Y/agouti-related peptide neuron [37, 38]. Recent basic study found GABAergic synapses to POMC neurons differentially responded to leptin at different levels of glucose [39]. We found in diabetic subjects that reduced HRV was significantly association of with duration of diabetes, even though it was not significantly associated with HbA1c level.

Second, the presence of neuropathy may explain a difference in diabetic patients. Some previous studies have addressed the association between leptin and diabetic neuropathy with controversial results. Serum leptin level tended to be associated with sensory conduction velocity [40] in diabetic patients, while another study did not find any relationship [41]. In our hand, plasma leptin and HRV were not significantly different between diabetic patients with and without neuropathy. Interestingly, a significant association of leptin with HRV was seen in patients with, but not those without neuropathy. On the other hand, a recent study observed a decrease in HRV even in individuals with newly detected diabetes as well as those with impaired glucose tolerance [42]. Thus, the presence of neuropathy may not sufficiently explain why leptin is significantly associated with HRV only in diabetic patients.

Third, other comorbidities differentially distributed in the present two groups, such as hypertension, eGFR, and adiposity, may influence and modulate HRV parameters [4, 43–45]. Indeed, a study in healthy subjects showed association of leptin with HRV is dependent on BMI levels [33]. Thus, significantly higher BMI level in diabetic patients (26.1 kg/m^2^) than in non-diabetic patients (23.3 kg/m^2^) might contribute to inconsistent relation of leptin with HRV in our study. Several studies have shown that cardiac autonomic function is impaired in patients with cardiovascular diseases [5, 6, 46]. However, the association between leptin and HRV was similar even after adjusting for previous CVD events, and the association remained similar in diabetic patients without previous CVD events.

### sOb-R and autonomic function

In this study, we also examined for the first time the association of plasma sOb-R with HRV. Although, plasma sOb-R tended to be inversely associated with adiposity, it was not significantly associated with any HRV parameters in our diabetic and non-diabetic patients.

### Limitations

Our study design was cross-sectional, thus, even though relationships were explored in predictive terms, the results cannot be interpreted for any causal relationships. Longitudinal follow-up examinations of this cohort will clarify the role of leptin in autonomic dysfunction in patients with and without diabetes.

## Conclusions

Our results showed that hyperleptinemia is associated with cardiac autonomic dysfunction in patients with diabetes, with the relationship independent of the accumulation of visceral fat.

## Abbreviations

Ob-R: receptor for leptin
HRV: heart rate variability
CVD: cardiovascular disease
DPP4: dipeptidyl peptidase 4
GLP1: glucagon-like peptide 1
PAD: peripheral arterial disease
eGFR: estimated glomerular filtration rate
CKD: chronic kidney disease
UAE: urinary albumin excretion
SDNN: standard deviation of the NN(RR) interval
SDANN5: standard deviation of the average NN(RR) intervals for each 5-min
sOb-R: soluble Ob-R
VFA: visceral fat area
SFA: subcutaneous fat area
BMI: body mass index
FPG: fasting plasma glucose
LF: low frequency
HF: high frequency
POMC: proopiomelanocortin
GABA: gamma-aminobutyric acid
